# Diverse functions of myosin VI in spermiogenesis

**DOI:** 10.1007/s00418-020-01954-x

**Published:** 2021-01-02

**Authors:** Przemysław Zakrzewski, Marta Lenartowska, Folma Buss

**Affiliations:** 1 Department of Cellular and Molecular Biology, Faculty of Biological and Veterinary Sciences, Nicolaus Copernicus University in Toruń, Torun, Poland; 2 Centre for Modern Interdisciplinary Technologies, Nicolaus Copernicus University in Toruń, Torun, Poland; 3 Cambridge Institute for Medical Research, The Keith Peters Building, University of Cambridge, Hills Road, Cambridge, CB2 0XY UK

**Keywords:** Actin, *C. elegans*, *Drosophila*, Mouse, Myosin VI, Spermiogenesis

## Abstract

Spermiogenesis is the final stage of spermatogenesis, a differentiation process during which unpolarized spermatids undergo excessive remodeling that results in the formation of sperm. The actin cytoskeleton and associated actin-binding proteins play crucial roles during this process regulating organelle or vesicle delivery/segregation and forming unique testicular structures involved in spermatid remodeling. In addition, several myosin motor proteins including MYO6 generate force and movement during sperm differentiation. MYO6 is highly unusual as it moves towards the minus end of actin filaments in the opposite direction to other myosin motors. This specialized feature of MYO6 may explain the many proposed functions of this myosin in a wide array of cellular processes in animal cells, including endocytosis, secretion, stabilization of the Golgi complex, and regulation of actin dynamics. These diverse roles of MYO6 are mediated by a range of specialized cargo-adaptor proteins that link this myosin to distinct cellular compartments and processes. During sperm development in a number of different organisms, MYO6 carries out pivotal functions. In *Drosophila*, the MYO6 ortholog regulates actin reorganization during spermatid individualization and male KO flies are sterile. In *C. elegans*, the MYO6 ortholog mediates asymmetric segregation of cytosolic material and spermatid budding through cytokinesis, whereas in mice, this myosin regulates assembly of highly specialized actin-rich structures and formation of membrane compartments to allow the formation of fully differentiated sperm. In this review, we will present an overview and compare the diverse function of MYO6 in the specialized adaptations of spermiogenesis in flies, worms, and mammals.

## Introduction

### Sequential stages of mammalian spermiogenesis

**Fig. 1:**
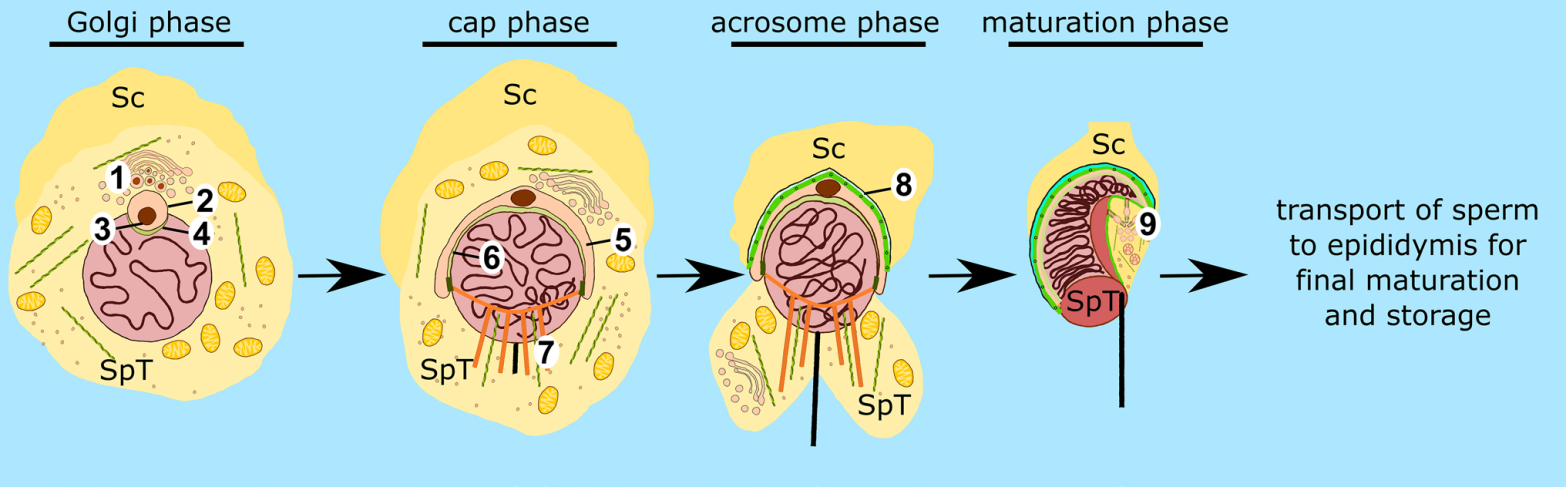
Schematic diagram highlighting the sequential steps of mouse spermiogenesis. During the Golgi phase, proacrosomal vesicles (*1*) fuse to form the acrosomal vesicle (*2*), which contains the acrosomal granule (*3*). The acrosomal vesicle adheres to the nuclear envelope through the acroplaxome (*4*). During the cap phase, the acrosomal vesicle flattens and spreads over the nucleus to form a cap (*5*). Acrosomal reshaping and spermatid elongation is mediated by the acroplaxome (*6*). During the acrosomal phase, the manchette (*7*) participates in the formation of the sperm flagellum. At this stage, spermatids are attached to Sertoli cells through the apical ES (*8*) which also supports their movement across the seminiferous tubule. Finally, during the maturation phase, the elongation of the spermatid is completed and most of the cytoplasm and organelles are removed and phagocytosed by Sertoli cells. The TBCs form at the spermatid-Sertoli cell interface (*9*) and internalize cell–cell junctions supporting the sperm release. *Sc* Sertoli cell, *SpT* spermatid

**Fig. 2:**
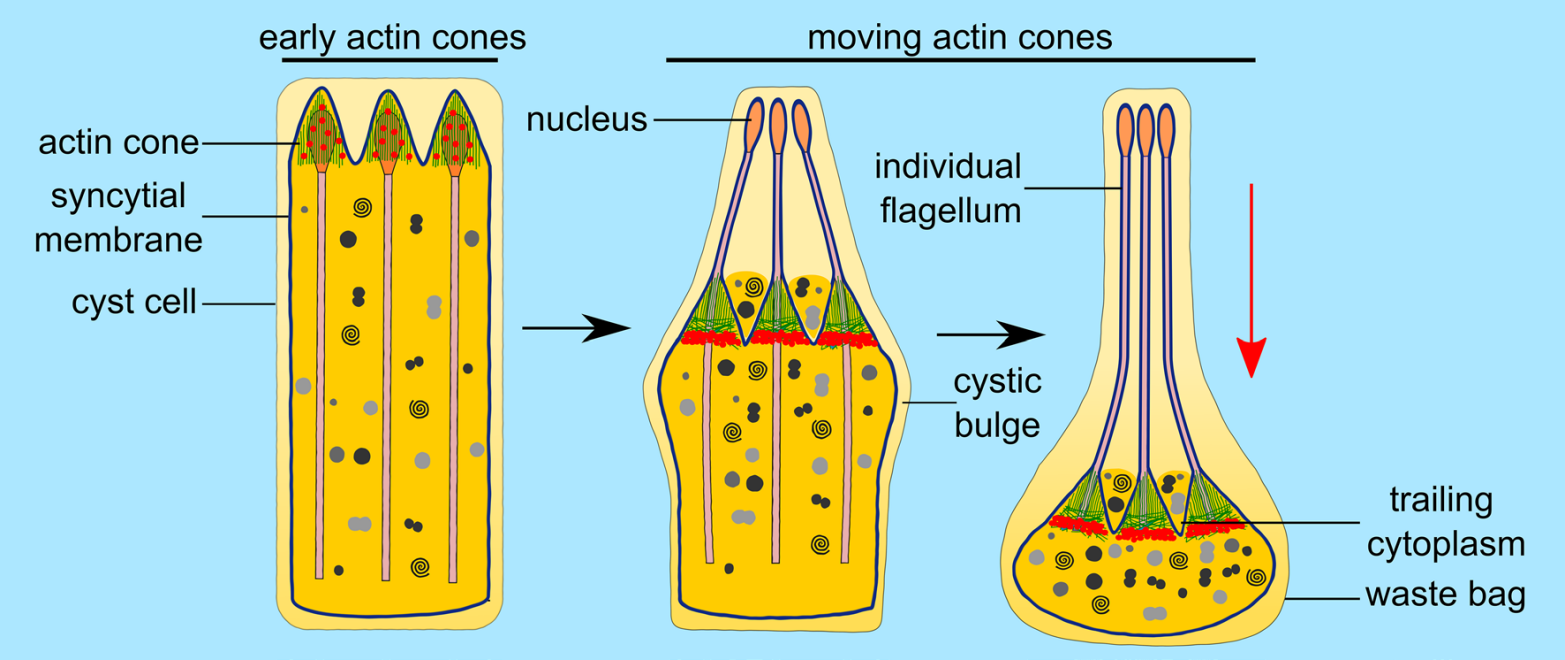
Spermatid individualization in *Drosophila*. In each testicular cyst a syncytial membrane is reorganized into individual membranes encasing 64 spermatids during individualization. This process is driven by actin cones, which assemble around nuclei of spermatids and move synchronously down the length of the axonemes. Early actin cones are made of parallel actin bundles and contain jaguar (ortholog of MYO6; *red dots*). As they move, the actin cones separate into two distinct domains—at the rear end parallel actin filament bundles predominate, whereas at the front actin filaments form a dense meshwork. At this stage, jaguar concentrates at the front of the actin cones. While the cytoplasm and organelles are extruded from spermatids, the cystic bulge forms. Remnants of the trailing cytoplasm can be observed between the moving actin cones. When the actin cones reach the end of the cyst, excess membrane and cytoplasm are pinched off in the form of the waste bag and the spermatids are left completely encased in individual membranes and with fully formed flagella. *Red arrow* shows the direction of movement of the actin cones

**Fig. 3:**
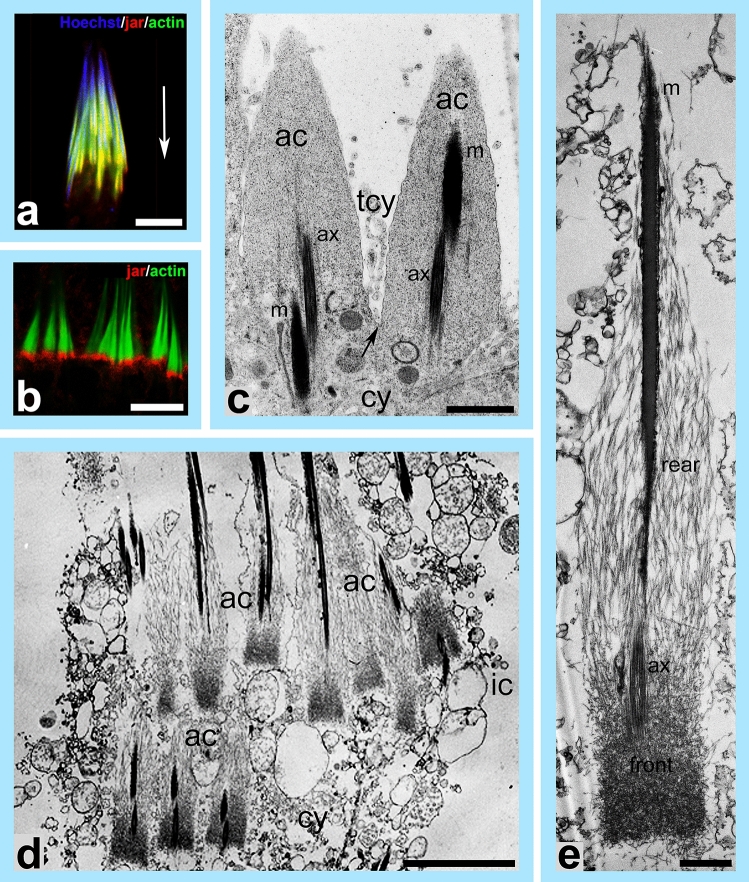
Localization of jaguar (MYO6 ortholog) and ultrastructure of *Drosophila* actin cones. **a** Localization of jaguar (*red*) by immunofluorescence in actin cones (*green*) at the beginning of spermatid individualization. Jaguar is present throughout the actin cones (*yellow* indicates overlap between jaguar and actin) that form around the nuclei of spermatids (*blue*). **b** Immunofluorescence localization of jaguar (*red*) in actin cones (visualized in *green*) at a later stage of spermatid individualization, when jaguar forms a dense band at the front of the moving actin cones. **c** Ultrastructural analysis of actin cones in a cyst isolated from *Drosophila* testis. *Arrow* indicates the syncytial membrane, which progresses downwards to separate the spermatids during individualization. **d** Ultrastructural visualization of actin cones in the cystic bulge decorated by myosin-II subfragment 1, which highlights the two distinct domains of the actin cones, a dense actin meshwork at the front and parallel actin filaments at the rear. The actin polymerization in the rear region drives cone movement and the actin meshwork at the front ensures exclusion of the cytoplasm and reorganization of the syncytial membrane. **e** High resolution electron microscope image of a single actin cone decorated by myosin-II subfragment 1. *ac* actin cone, *ax* axoneme, *cy* cytoplasm, *ic* individualization complex, *m* mitochondrion, *tcy* trailing cytoplasm. *White arrow* in ***a ***shows the direction of actin cones movement, which is the same for **b**–**e**. *Bars* 5 µm (**a**–**b**, **d**), 1 µm (**c**, **e**)

**Fig. 4:**
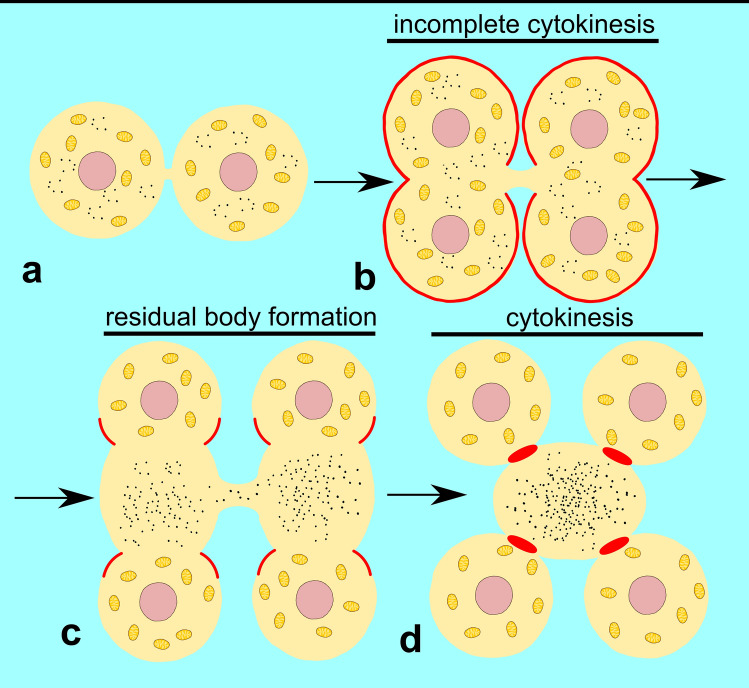
Model of spe-15 (MYO6 ortholog) function during spermatid differentiation in *C. elegans.* After meiosis, two haploid spermatids remain connected and differentiate by shedding residual cytoplasm in the form of residual body. Spermatid budding is mediated by spe-15 (*red*). Mitochondria are transported to spermatids, whereas all ribosomes and remaining organelles are packed to the residual body. Spermatids detach from the residual body following the cytokinesis mediated by spe-15 and next mature into spermatozoa (based on Hu et al. 2019a, b)

**Fig. 5:**
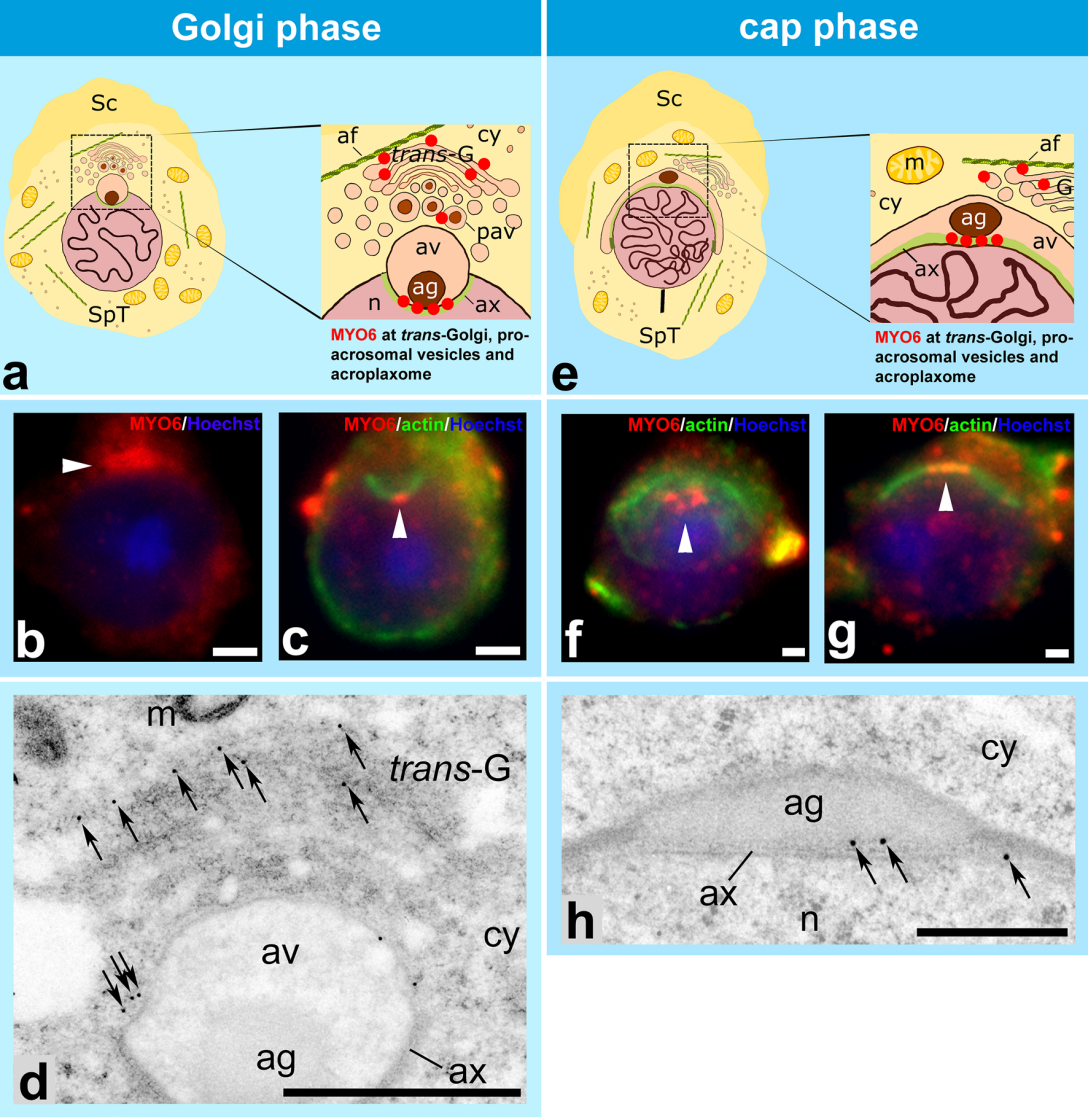
Localization of MYO6 in mouse developing spermatids during the Golgi and cap phases. **a** During the Golgi phase, MYO6 (*red dots*) localizes to the *trans-*Golgi network, proacrosomal vesicles and acroplaxome below the acrosomal granule. **b, c** Immunofluorescence localization of MYO6 (*red*) at the Golgi complex (**b**) and at the acroplaxome (*green, c).* *Arrowhead* indicates the Golgi complex in *(b)* and the area below the acrosomal granule in (**c**). **d** Ultrastructural localization of MYO6 using immunogold labeling at the *trans*-Golgi network and on the surface of the acrosomal vesicles (*arrows*). **e** During the cap phase, MYO6 (*red dots*) localizes to the *trans-*Golgi network, proacrosomal vesicles and acroplaxome below the acrosomal granule. **f** and **g** Immunofluorescence localization of MYO6 (*red*) at the acroplaxome (*green*). *Arrowheads* indicate area below the acrosomal granule. **h** Ultrastructural localization of MYO6 using immunogold labeling at the acroplaxome below the acrosomal granule (*arrows*). Panel **d **is modified from Zakrzewski et al. (2017) (published under CC BY 4.0). *af* actin filament, *ag* acrosomal granule, *av* acrosomal vesicle, *ax* acroplaxome, *cy* cytoplasm, *m* mitochondrion, *n* nucleus, *pav* proacrosomal vesicles, *trans-G* *trans*-Golgi network, *Sc* Sertoli cell, *SpT* spermatid. *Bars* 1 µm

**Fig. 6:**
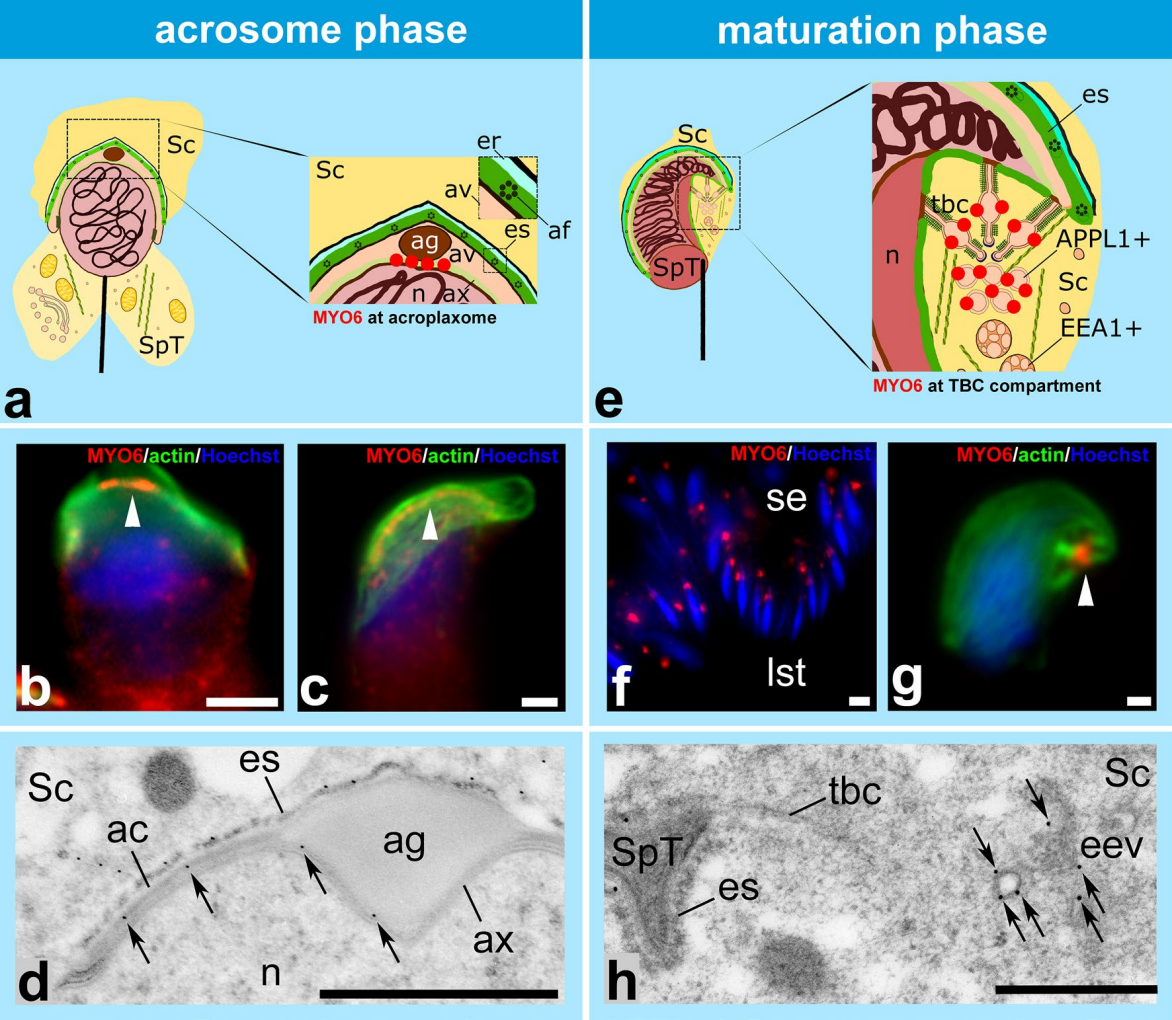
Localization of MYO6 in mouse developing spermatids during the acrosome and maturation phases. **a** During the acrosome phase, MYO6 (*red dots*) localizes to the acroplaxome below the acrosomal granule. **b, c** Immunofluorescence localization of MYO6 (*red*) at the acroplaxome (actin visualized in *green*) in elongating spermatids during the acrosome phase. *Arrowheads* indicate area below the acrosomal granule. **d** Ultrastructural localization of MYO6 using immunogold labeling at the acroplaxome below the acrosomal granule and the acrosome (*arrows*). Panel **d** is modified from Zakrzewski et al. (2017) (published under CC BY 4.0). **e** During the maturation phase, MYO6 (*red dots*) is concentrated at the bulbs of the TBCs and APPL1-positive early endosomes. **f** Immunofluorescence localization of MYO6 (*red*) at the spermatid-Sertoli cell interface in the seminiferous epithelium on a semi-thin paraffin section. During this stage, maturing spermatids are close to the lumen of the seminifereous tubules. **g** Immunofluorescence localization of MYO6 (*red*) in the endocytic compartment of the TBCs (actin visualized in *green*). **h** Ultrastructural localization of MYO6 using immunogold labelling in early endosomes in a spermatid during the maturation phase (*arrows*). *ac* acrosome, *af* actin filament, *ag* acrosomal granule, *APPL1* + APPL1-positive early endocytic vesicle, *av* acrosomal vesicle, *ax* acroplaxome, *EEA1* + EEA1-positive early endosome, *eev* early endocytic vesicle, *er* endoplasmic reticulum, *es* apical ES, *lst* lumen of seminiferous tubule, *n* nucleus, *Sc* Sertoli cell, *se* seminiferous epithelium, *SpT* spermatid, *tbc* tubulobulbar complex. *Bars* 5 µm (**f**), 1 µm (**b**–**d**, **g**), 500 nm (**h**)

**Fig. 7:**
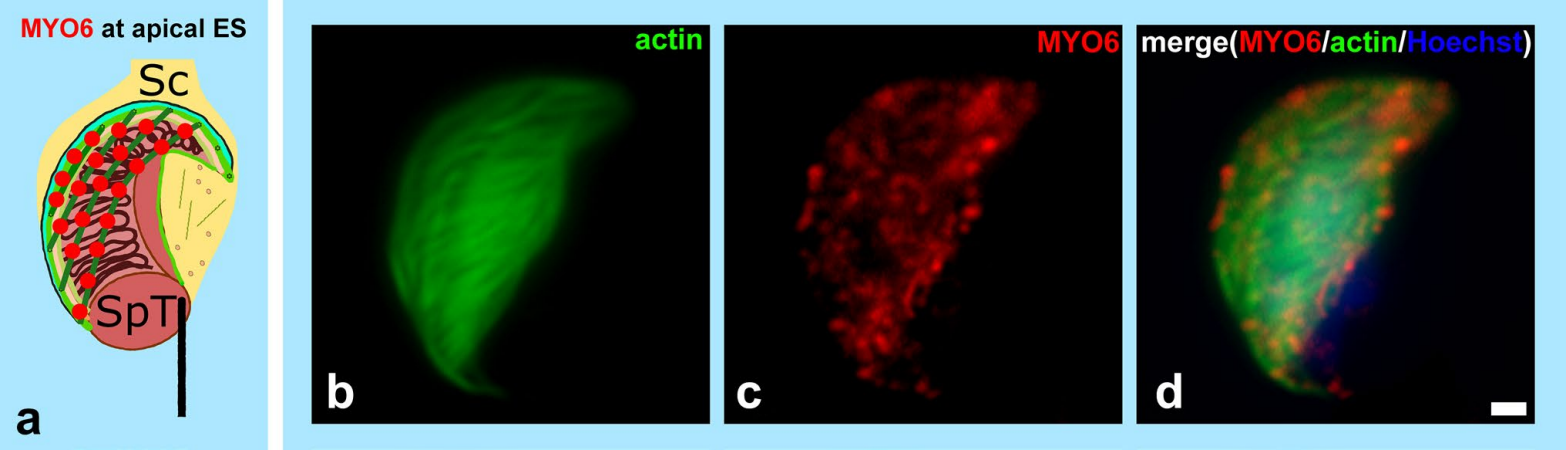
MYO6 localizes to the apical ES. During the maturation phase, MYO6 (*red*) is also present at the apical ES, where it localizes to actin bundles (visualized in **b** and **d** in *green*) that enclose the maturing spermatids. *Sc* Sertoli cell, *SpT* spermatid. *Bars* 1 µm

**Fig. 8:**
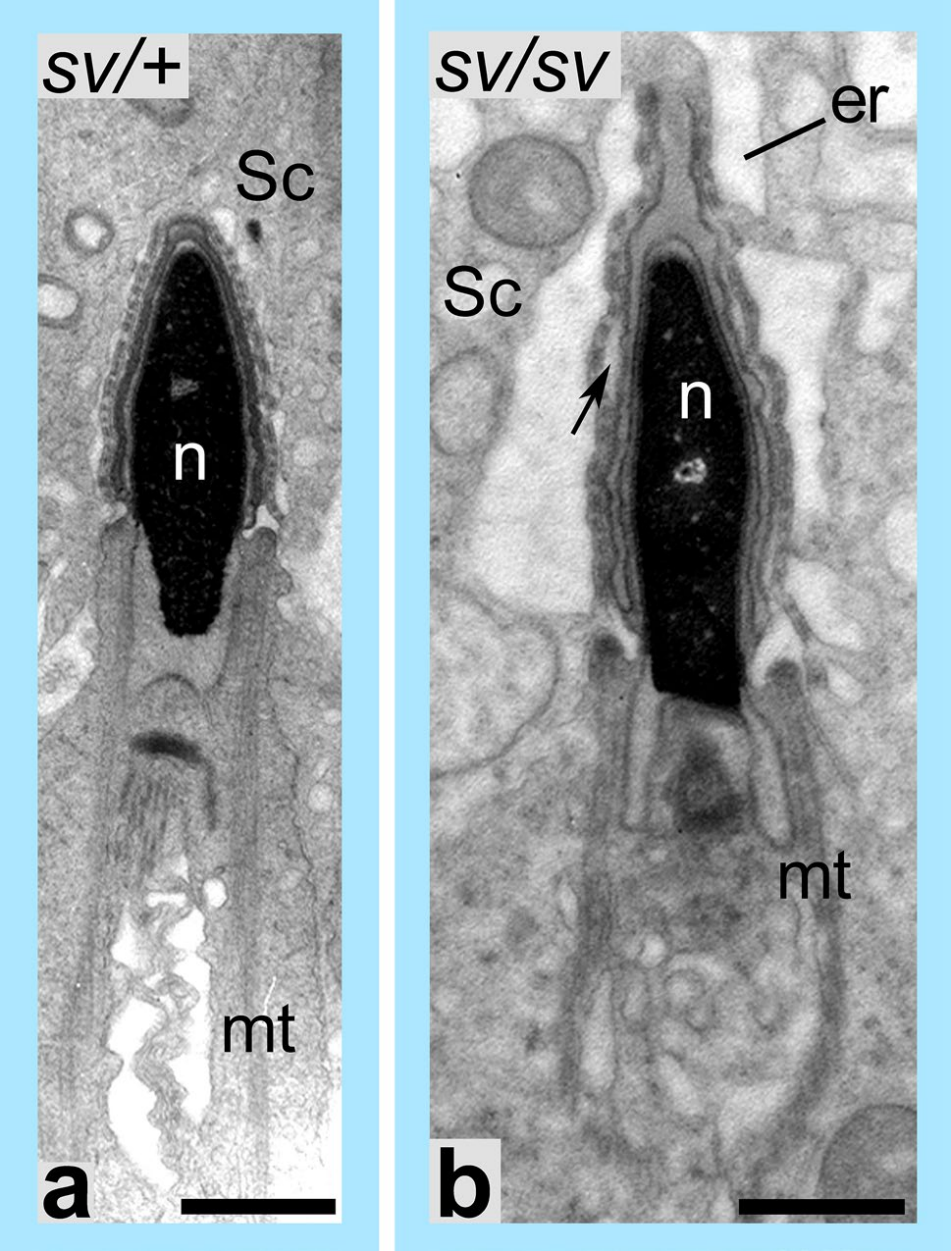
Loss of MYO6 causes ultrastructural disruptions of the apical ES in maturing spermatids. Ultrastructural analysis of the apical ES of a MYO6-expressing spermatid (**a**) and MYO6-deficient spermatid (**b**). In the absence of MYO6 the apical ES appears to be disrupted and detachment of the spermatid head from the apical ES and swelling of the ER can be observed (*arrow*) (**b**). *er* endoplasmic reticulum, *mt* manchette, *n* nucleus, *Sc* Sertoli cell. *Bars* 1 µm

Spermiogenesis is the final stage of spermatogenesis, an evolutionally conserved differentiation process that results in the formation of mature spermatozoa. In mammals, spermiogenesis can be divided through microscopic observations into four distinct phases: the Golgi, the cap, the acrosome, and the maturation phase (Toshimori 2009); (Fig. 1). During the Golgi phase, proacrosomal vesicles (Fig. 1, *1*) derived from the trans-Golgi network and the endocytic pathway fuse to form the acrosomal vesicle (Fig. 1, *2*), which contains the acrosomal granule (Fig. 1, *3*). The acrosomal vesicle adheres through a cytoskeletal plate called the acroplaxome (Fig. 1, *4*) to the nuclear envelope. As demonstrated in rodents, the acroplaxome consists of actin filaments and the intermediate filament protein, SAK57 (spermatogenic cell/sperm-associated keratin of molecular mass 57 kDa), which forms the marginal ring terminating the acroplaxome and connecting, together with additional linker proteins, the acrosome to the nuclear lamina (Kierszenbaum et al. 2003a). During the cap phase, the acrosomal vesicle flattens out and spreads over the spermatid nucleus to form a cap (Fig. 1, *5*); (Toshimori 2009). This process of acrosomal reshaping and spermatid elongation is mediated by the flexible F-actin scaffold of the acroplaxome (Fig. 1, *6*); (Kierszenbaum et al. 2003a, 2011). During the acrosomal phase, another transient structure, the manchette, is formed (Fig. 1, *7*), which consists of a perinuclear ring, and contains actin filaments and microtubules that extend into the elongating sperm tail (Kierszenbaum et al. 2002, 2003a, 2007). The manchette is involved in sperm head shaping and protein transport along the cytoskeleton important for formation of the sperm flagellum. Finally, the elongation of spermatids is completed during the maturation phase, and most of the spermatid cytoplasm and organelles are discarded in the form of residual bodies that are phagocytosed by Sertoli cells. Two additional testis-specific structures are formed during spermiogenesis in mammals—the apical ectoplasmic specialization (apical ES); (Fig. 1, *8*) and the tubulobulbar complexes (TBCs); (Fig. 1, *9*). The apical ES is a specialized actin-rich structure formed between spermatids and Sertoli cells and is composed of parallel actin bundles sandwiched between the spermatid plasma membrane and the Sertoli cell’s endoplasmic reticulum (ER); (Toyama 1976; Russell 1977; Franke et al. 1978; Sun et al. 2011). The apical ES anchors developing spermatids to the Sertoli cells, positions the spermatid head regions in the correct orientation, and supports the spermatid movement across the seminiferous tubule. TBCs are also assembled at the spermatid–Sertoli cell interface and are involved in the internalization of cell–cell junctions to facilitate sperm release (Upadhyay et al. 2012; Vogl et al. 2013). TBCs consist of a long endocytic proximal tubule stabilized and cuffed by a dense actin meshwork, an actin-free swollen bulbular region surrounded by endoplasmic reticulum, and a short distal tubule terminating in a clathrin-coated pit (Russell and Clermont, 1976; Russell 1979; Vogl et al. 1985). After the reorganization of the apical ES and internalization of cellular attachments via the TBCs, spermatogenesis ends with sperm release to the lumen of seminiferous tubules in a process called spermation. The still non-motile sperm is transported to the epididymis, where the major part of the maturation process occurs, before the final capacitation to acquire hypermotility that takes place within the female reproductive tract (Skerget et al. 2015). Moreover, some of the luminal components of the epididymis, which are crucial for final sperm maturation, such as the androgen binding protein, transferrin, and immobilin, are also endocytosed and recycled by the microvillar epididymal epithelium (Zhou et al. 2018).

### Actin cytoskeleton in spermiogenesis

Directly after meiosis, haploid spermatids enter spermiogenesis as round unpolarized cells that undergo excessive remodeling leading to the formation of mature sperm. In mammals, the actin cytoskeleton plays an undisputed role at several key points during this process serving as a cytoskeletal track to guide exocytic vesicles from the Golgi to the acrosome or from the manchette to the centrosome/axoneme. In addition, actin filaments are crucial for the assembly and remodeling of testis-specific structures important for spermatid development, including the acrosome–acroplaxome–manchette complex, the apical ES, and the TBCs (Lie et al. 2010b; O’Donnell et al. 2011; Upadhyay et al. 2012; Qian et al. 2014a, b; Dunleavy et al. 2019; Pleuger et al., 2020; Yang and Yang 2020). Actin dynamics is spatiotemporally regulated by different actin-binding proteins (ABPs) and some of these actin regulators have been shown to be involved in mammalian spermiogenesis. Localization data in rat testes are available for a number of ABPs, such as espin, fimbrin, vinculin, plastin-3, and EPS8 (epidermal growth factor receptor pathway substrate 8), that are present at the apical ES (Grove and Vogl 1989; Grove et al. 1990; Bartles et al. 1996; Chen et al. 1999; Lie et al. 2009; Siu et al. 2011; Li et al. 2015) whereas the highly branched actin filament network of TBCs has been shown to associate with different actin regulators including cofilin, cortactin, N-WASP (neuronal Wiskott-Aldrich syndrome protein), and ARP3 (actin-related protein 3) both in rats and mice (Guttman et al. 2004a; Young et al. 2009; Lie et al. 2010a). Furthermore, cortactin regulates the acrosome–acroplaxome–manchette complex dynamics and tyrosine phosphorylated cortactin has been localized to the rat acroplaxome, while non-phosphorylated cortactin to the manchette (Kierszenbaum et al. 2008). Finally, a number of actin-based myosins are involved at different stages of sperm development in mammals (Li and Yang 2016). Myosin Va (MYO5a), for example, plays a role in acrosome biogenesis in rodents by transporting proacrosomal vesicles along actin filaments and facilitating their fusion to the acroplaxome (Kierszenbaum et al. 2003b, 2004). Myosins of class V have also been suggested to be involved in the intra-manchette transport for the delivery of cargo to the centrosome in the developing sperm flagellum during human spermiogenesis (Hayasaka et al. 2008). Myosin VIIa (MYO7a) has been implicated in the adhesion and transport of developing spermatids across the rat seminiferous epithelium and its depletion perturbs the spatiotemporal expression of different ABPs involved in spermiogenesis (Velichkova et al. 2002; Wen et al. 2019). Finally, gene expression profiling in rodent tissues has revealed that transcripts of the minus-directed myosin VI (MYO6) are present in mouse testes (Avraham et al. 1995) and that two different MYO6 isoforms are expressed in mouse and rat testes (Buss et al. 2001; Zakrzewski et al. 2017).

### Structure and function of MYO6

Myosin motor proteins translocate along actin filaments, form dynamic tethers between the actin cytoskeleton and membrane compartments or regulate actin filament organization and dynamics. MYO6 is a highly unusual myosin motor, which unlike other myosins moves towards the minus end of actin filaments (Wells et al. 1999). This specialized feature of MYO6 may explain the many proposed functions of this myosin in a wide array of cellular processes in animal cells. Overall, the structure of MYO6 follows the general domain organization of other proteins in the myosin family: an N-terminal motor domain (head) which binds actin filaments and ATP and converts biochemical energy into mechanical force, a neck region (lever arm) that binds light chains or calmodulins, which regulate motor properties and a tail domain important for dimerization and cargo-binding and precise spatiotemporal targeting. In addition, MYO6 has two unique inserts: the first one in the motor domain near the ATP-binding pocket may regulate ATPase activity, while the second insert, also called the “reverse gear”, between the motor domain and the lever arm is responsible for the reverse reposition of the lever arm at the end of the power stroke allowing its “backward” movement (Menetrey et al. 2005). Finally, the MYO6 tail is alternatively spliced at two sites giving rise to four different tissue-specific MYO6 variants with either a large insert (LI, 12–32 amino acids) or a small insert (SI, 9 amino acids), no insert or both inserts (LI + SI); (Buss et al. 2001; Dance et al. 2004; de Jonge et al. 2019). These inserts regulate the binding of different adaptor proteins and thus determine the subcellular localization and function of MYO6 in different cell types and tissues (Buss et al. 2001; Wollscheid et al. 2016; O’Loughlin et al. 2018).

To date, MYO6 has been implicated in a number of cellular processes including endocytosis, secretion, stabilization of the Golgi complex, autophagy, mitophagy, regulation of actin dynamics, myogenesis, and transcription (Buss et al. 2001; Warner et al. 2003; Sahlender et al. 2005; Tumbarello et al. 2012, 2013, 2015; Tomatis et al. 2013, 2017; Karolczak et al. 2015b; Fili et al. 2017; Kruppa et al. 2018; Majewski et al. 2018; O’Loughlin et al. 2018; de Jonge et al. 2019). One of the key factors that regulates the function of MYO6 in these diverse processes is its ability to bind different adaptor proteins within the cargo-binding domain in the tail. For instance, via interaction with the clathrin and endocytic adaptor protein DAB2 (Disabled-2), the LI isoform of MYO6 is targeted to clathrin-coated pits/vesicles, where it facilitates receptor uptake at the apical domain of polarized epithelial cells (Morris et al. 2002; Wollscheid et al. 2016). In contrast, the MYO6 NI isoform interacts with GIPC1 (GAIP C-terminus-interacting protein) and TOM1/L2 (Target of Myb protein 1/Target of Myb-like protein 2), which target MYO6 to APPL1-(Adaptor Protein, Phosphotyrosine Interacting With PH Domain And Leucine Zipper 1) and RAB5-(Ras-related in brain) positive early endosomes and regulate translocation of these early endosomes through the dense actin cortex below the plasma membrane, which facilitates their maturation and regulates downstream endosomal signaling (Aschenbrenner et al. 2003; Tumbarello et al. 2012, 2013; Masters et al. 2017; O’Loughlin et al. 2018; de Jonge et al. 2019). Further MYO6-binding proteins are optineurin, NDP52 (nuclear dot protein 52 kDa), and TAX1BP (Tax1-binding protein 1), which are selective autophagy receptors and are believed to link MYO6 function to autophagosome maturation (Sahlender et al. 2005; Morriswood et al. 2007; Tumbarello et al. 2013, 2015). At present, it is not known whether MYO6 operates in these diverse cellular processes as a cargo transporter or as a protein/organelle anchor. Finally, MYO6 has recently been linked to several RhoGEF complexes, which suggests an active role in modulating actin track dynamics as well as in regulating septin organization. MYO6, for example, has been identified in a complex with LRCH3 (leucin-rich repeat and calponin homology domain-containing protein 3) and DOCK7 (Dedicator of cytokinesis protein 7), a GEF for RAC (Ras-related C3 botulinum toxin substrate) and CDC42 (cell division control protein 42 homolog), and in a complex with GIPC1 and LARG (Leukemia-associated Rho guanine nucleotide exchange factor), a RhoGEF (O’Loughlin et al. 2018; de Jonge et al. 2019).

The Snell’s waltzer (*sv/sv*) mouse contains a spontaneous mutation in the *Myo6* gene, which leads to the complete absence of MYO6 in the homozygous mouse (Avraham et al. 1995). Snell’s waltzer mice are deaf and exhibit a tail-chaser phenotype due to vestibular dysfunction that results from the neurosensory epithelium degeneration in the inner ear (Deol and Green 1966; Avraham et al. 1995; Self et al. 1999; Roux et al. 2009). These mice also display several other defects in a variety of tissues and organs, such as aberrations in the Golgi morphology, reduced secretion, defective endocytosis, and impaired morphology of brush border enterocytes and hippocampal neurons (Warner et al. 2003; Osterweil et al. 2005; Ameen and Apodaca 2007; Collaco et al. 2010; Gotoh et al. 2010; Hegan et al. 2012, 2015b). Moreover, profound fibrosis and both cardiac and pulmonary vascular endothelial defects were observed in MYO6 mutant mice (Hegan et al. 2015a). Interestingly, not only in vertebrates but also in *Drosophila* depletion of the MYO6 ortholog, jaguar, leads to a variety of abnormal phenotypes, especially during embryogenesis, and interestingly, spermiogenesis (Mermall et al. 1995; Deng et al. 1999; Hicks et al. 1999; Millo et al. 2004).

## Diverse functions of MYO6 in spermiogenesis

### MYO6 in Drosophila spermatid maturation

At the final step of *Drosophila *spermatogenesis called spermatid individualization, a cyst of 64 syncytial spermatids is reorganized into individual sperm by membrane remodeling and removal of cytoplasmic content (Tokuyasu et al. 1972; Noguchi and Miller 2003). This process is driven by long-lived actin structures, so-called actin cones, which assemble around spermatid nuclei and travel synchronously along the axonemes to the ends of the tails (Fig. 2). As they move, most of the spermatid cytoplasm is pushed out of the flagellum, accumulating in the cystic bulge, and finally discarded in the form of a waste bag. At the same time, the cyst membrane is reorganized into individual sperm membranes (Fig. 3c; *arrow* shows a membrane connecting actin cones, which is then pulled over individual spermatids). Interestingly, newly formed actin cones are composed exclusively of actin bundles, whereas moving cones develop two domains—a rear region of parallel bundles and a dense actin meshwork at the front (Fig. 3d, e); (Noguchi et al. 2006, 2008). In wild-type *Drosophila *testes, at the beginning of sperm individualization, jaguar, also called myosin heavy chain 95F (encoded by *jar*) is present throughout the actin cones, whereas later, when the individualization cones move away from the spermatid nuclei, it concentrates at the front of the actin filament meshwork (Fig. 3a, b). In jaguar-depleted (*jar*^*1*^*/jar*^*1*^*)* flies actin filament assembly is disrupted causing loss of the actin meshwork at the front of the cone and as a result the movement of the actin cones stops before individualization is completed (Hicks et al. 1999; Rogat and Miller 2002; Noguchi et al. 2006; Lenartowska et al. 2012). Cellular components are no longer removed from mutant spermatids and sperm tails are not always separated by individual plasma membranes. All these cellular disruptions lead to the cessation of the individualization process and male infertility, which highlights a crucial role for jaguar during *Drosophila* spermiogenesis (Noguchi et al. 2006).

Polarized distribution of selected ABPs in moving *Drosophila* actin cones has also been demonstrated. The actin nucleating complex, ARP2/3, and its activator, cortactin, are strongly enriched in the dense actin meshwork at the front of the cone, while two actin-bundling proteins, quail (a villin ortholog) and singed (a fascin ortholog), are localized at the rear end of the cone (Rogat and Miller 2002). Interestingly, when the ARP2/3 is absent, the cones still move but meshwork formation is compromised, and membrane reorganization and cytoplasmic exclusion are abnormal, and individualization fails. In contrast, when profilin (a regulator of actin assembly) is absent, bundle formation is greatly reduced, the meshwork still forms but no movement occurs (Noguchi et al. 2008). Thus, the two different cone domains at the front and rear are differentially regulated and have different functions during spermatid individualization: the bundles stabilized by actin cross-linkers at the rear are required for the cone movement, whereas the actin meshwork formed by ARP2/3 at the cone front is involved in removing the cytoplasm from the sperm tails. Interestingly, in *jar*^*1*^*/jar*^*1*^ testes, the distribution of selected ABPs is disrupted and the specific localization of MYO6 at the front of the cone maintains its shape and size (Rogat and Miller 2002; Isaji et al. 2011). These findings indicate that jaguar stabilizes actin cone structure and plays an anchoring role during *Drosophila* spermiogenesis by tethering different cargo/membranes to actin filament structures. Although the precise molecular mechanism of jaguar function in this process is still unknown, both the motor domain and cargo-binding tail domain are required for intracellular targeting of jaguar and dense meshwork formation at the front of the cone during *Drosophila* spermiogenesis (Noguchi et al. 2006; Isaji et al. 2011). The correct targeting and function of jaguar require the conserved RRL and LWY motif responsible for binding molecular partners as well as the WKA motif that binds PtdIns(4,5)P2, which indicates that adaptor protein as well as lipid binding are required for jaguar function at the front of moving actin cones (Isaji et al. 2011). Unfortunately, the jaguar adaptor proteins involved in this process in *Drosophila* have not been identified so far.

### Proposed function of MYO6 in *C. elegans* spermatid differentiation

Morphological rearrangements in nematode spermatogenesis do not lead to the formation of flagellated sperm, but instead give rise to spermatozoa that use amoeboid motility. In *C. elegans*, the differentiation of haploid spermatids into mature spermatozoa involves the asymmetric segregation of cellular material (Fig. 4); (Kelleher et al. 2000). While mitochondria and specialized Golgi-derived organelles are sorted into the developing sperm, other surplus organelles and components, such as ribosomes, actin filaments and microtubules, are removed from the spermatids and deposited in the residual body (RB). This asymmetric division of cytosolic content, the RB formation to collect the excluded material, followed by spermatid release from the RB through cytokinesis, involves two myosin motors, NMY2, the ortholog of human non-muscle myosin II (NMII, encoded by *nmy-2*) and spe-15, the ortholog of human MYO6 (encoded by *spe-15*) (Kelleher et al. 2000; Hu et al. 2019a). NMY2 drives an incomplete cytokinesis by forming an actomyosin ring that initiates cleavage furrow ingression, however, fails to complete restriction. The NMY2-mediated incomplete cytokinesis is believed to provide the force for RB expansion. Spe-15, in contrast, assembles into an actin-spe-15 ring, which constricts the membrane between the spermatid and RB and finally causes spermatid budding through spe-15-dependent cytokinesis. This process is dependent on the spe-15 adaptor protein GIPC (encoded in *C. elegans* by *gipc-1*), which may indicate that dimerization/multimerization of MYO6 is required for cytokinesis and spermatid release. Depletion of NMY2 or spe-15 causes defects in the asymmetric segregation of cytosolic components.

### MYO6 in mammalian spermiogenesis

#### Localization of MYO6 in mouse testes

MYO6 is broadly expressed in different animal tissues including the testes in humans, rodents, worms and *Drosophila* (Kelleher et al. 2000; Kellerman and Miller 1992; Hasson and Mooseker 1994; Avraham et al. 1995, 1997). Moreover, PCR analysis demonstrated that two MYO6 isoforms (the SI and NI) are expressed in rodent testes (Buss et al. 2001; Zakrzewski et al. 2017) and are associated with several key actin-rich structures throughout sperm development and maturation in mice (Figs. 5 and 6); (Zakrzewski et al. 2017, 2020a, b). During the Golgi phase, MYO6 is present at/around the Golgi complex adjacent to the acrosome-nuclear pole, including the *trans-*Golgi network and uncoated and as well as coated vesicles and at the inner acrosome membrane–acroplaxome interface (Fig. 5a–d); (Zakrzewski et al. 2017, 2020a). During the cap phase, MYO6 continues to be present at the *trans-*Golgi network and surrounding vesicles and at the acroplaxome, especially directly below the electron-dense acrosomal granule (Fig. 5e–h); (Zakrzewski et al. 2017, 2020a). During the following phase, the acrosome or elongation phase, when the acrosome spreads over the spermatid nucleus, MYO6 is still present at the acroplaxome (Fig. 6a–d); (Zakrzewski et al. 2017, 2020a). Finally, during the maturation phase, MYO6 is found at TBCs, predominantly at the bulbular region and at an early endocytic compartment (Fig. 6e–h); (Zakrzewski et al. 2020b). In the following sections, we will describe how the loss of MYO6 impacts on the organization of the actin cytoskeleton and specialized membrane compartments during the different stages of mammalian spermiogenesis in the Snell’s waltzer mouse.

#### Loss of MYO6 causes morphological changes during acrosome biogenesis in mouse

##### Defects in Golgi organization and vesicle trafficking

Several membrane trafficking routes have been proposed to be involved in acrosome formation, including the transport of proacrosomal vesicles from the *trans*-Golgi network to the developing acrosome in the secretory pathway (Figs. 1 and 5a), the direct transport from the plasma membrane along the endocytic pathway or directly from lysosomes to the acrosome (Clermont and Tang 1985; Toshimori 1998, 2009; Kierszenbaum et al. 2003a). Thus, acrosome biogenesis includes the dynamic flow of vesicles at the intersection of exocytic and endocytic membrane trafficking routes (Raposo et al. 2007; Berruti et al. 2010; Berruti and Paiardi 2011; Delevoye et al. 2019). Indeed, the importance of the secretory pathway in acrosome biogenesis is highlighted by the finding that lack of GOPC (Golgi-associated PDZ- and coiled-coil motif-containing protein), which is involved in vesicle transport from the Golgi complex in other cell types, inhibits acrosome formation in developing mouse spermatids (Yao et al. 2002). GOLGA3 (Golgin subfamily A member 3) and PICK1 (protein interacting with C kinase 1), which both have been shown to interact with GOPC, are also present at the Golgi complex in developing spermatids and thus may contribute to secretory vesicle formation and delivery during acrosome development (Bentson et al. 2013; Xiao et al. 2009). Proacrosomal vesicles may be transported via two different cytoskeletal routes: along actin filaments, involving unconventional MYO5a or along microtubules with the help of KIFC1 (Kinesin family member C1); (Kierszenbaum et al. 2003b, 2004, 2011; Yang and Sperry 2003). In addition, our recent results have shown that also MYO6 plays a role during the early stages of acrosome biogenesis. Ultrastructural analysis of developing Snell’s waltzer spermatids revealed several defects affecting acrosome formation, including partial disruption of the Golgi complex and impairment of proacrosomal vesicular trafficking (Zakrzewski et al. 2020a). A more detailed analysis of the Golgi morphology in *sv/sv* spermatids demonstrated different morphological phenotypes—the asymmetrical orientation of the Golgi complex in the relation to the upper pole of spermatid nucleus and the loss of Golgi integrity (Zakrzewski et al. 2020a). Enlarged and swollen Golgi stacks and vesicles were observed in *sv/sv *spermatids, similar to those seen in cells treated with filamentous-actin-depolymerizing agents (Egea et al. 2013, 2015). Interestingly, MYO6 is present at the Golgi complex in different cell types and its depletion has been shown to cause changes in Golgi morphology, including its fragmentation, elongation, and reduction in size (Buss et al. 1998; Warner et al. 2003; Sahlender et al. 2005; Puri et al. 2010; Majewski et al. 2011; Karolczak et al. 2015a). Therefore, it is tempting to speculate that also in mouse testes MYO6 may play an anchoring role during spermiogenesis linking Golgi membranes to the surrounding actin filaments to maintain its morphology and position close to the developing acrosome.

The two splice variants of MYO6, the NI and SI isoforms, which are both expressed in mammalian testes, are not only important for exocytosis and the transport and tethering of secretory vesicles but the NI isoform of MYO6 is also present at early endosomes, where it is involved in the early stages of endocytosis (Buss et al. 2001; Aschenbrenner et al. 2003; Dance et al. 2004; Au et al. 2007; Chibalina et al. 2007; Inoue et al. 2008; Puri 2009, 2010; Majewski et al. 2010; Bond et al. 2012; Tumbarello et al. 2012; Tomatis et al. 2013) Therefore, it is tempting to hypothesize that MYO6 may participate in the transport of exocytic vesicles from the Golgi complex and/or endocytic vesicles to the developing acrosomal vesicle.

At present, however, we have very little insight into the exact function of MYO6 during acrosome biogenesis. MYO6 could be involved in short-range transport of proacrosomal vesicles in the opposite direction to MYO5a. Alternatively, MYO6 could be involved in the tethering of proacrosomal vesicles to the surrounding actin cytoskeleton. In mouse spermatids, during acrosome biogenesis, the MYO6-binding partner TOM1/L2 is present on vesicular structures located between the *trans*-Golgi network and maturing acrosome (Zakrzewski et al. 2020a). TOM1/L2 is a monomeric protein, which forms a 1:1 complex with MYO6 and does not induce dimerization or multimerization of MYO6 in contrast to other binding partners, such as DAB2 or GIPC1 (Yu et al. 2009; Shang et al. 2017; Hu et al. 2019b). Thus, the monomeric MYO6 in complex with TOM1/L2 may perform a tethering function, in contrast to a dimeric/multimeric myosin, which can move processively over short distances. Although the exact cellular function of the MYO6-TOM1/L2 complex remains to be established, both proteins are present on early endosomes and facilitate the delivery of these endosomes to autophagosomes required for their maturation and fusion with lysosomes (Tumbarello et al. 2012; O’Loughlin et al. 2018; de Jonge et al. 2019). Indeed, another observation in Snell’s waltzer spermatids suggests that the absence of MYO6 may affect the fusion of proacrosomal vesicles with the developing acrosome, as the number of proacrosomal vesicles appears to be elevated (Zakrzewski et al. 2020a). A similar phenotype was observed in mice depleted of ATG7 (autophagy-related protein 7), which highlights a potential involvement of the autophagy pathway in acrosome biogenesis (Wang et al. 2014). These observations support the notion that in mouse testes, the MYO6-TOM1/L2 complex may support tethering of proacrosomal vesicles in the vicinity of the upper pole of the spermatid nucleus to facilitate fusion between the proacrosomal vesicles and the developing acrosome (Zakrzewski et al. 2020a). However, further research is required to establish the exact function of MYO6 during acrosome biogenesis in mammals and to determine the polarity of actin filaments around proacrosomal vesicles, the Golgi complex, and the acrosome, which directs the minus-end-directed movement of MYO6.

##### Loss of acrosome symmetry

The fusion of proacrosomal vesicles leads to the formation of the glycoprotein-rich acrosomal vesicle, which gradually spreads over the spermatid nucleus forming a cap (Figs. 1 and 5a, e); (Toshimori et al. 2009). Both proacrosomal and acrosomal vesicles contain an electron-dense core with an amyloidogenic structure called the acrosomal granule, which is enriched in acidic hydrolytic enzymes (Khawar et al. 2019). This granule closely associates with the inner acrosomal membrane at the center of the acroplaxome. As the acrosomal vesicle flattens, the electron-dense material of the granule fills the entire acrosomal matrix (Toshimori et al. 2009). Similar to dense-core secretory granules, the low intraluminal pH of the acrosomal vesicle may drive the concentration and compartmentalization of acrosomal proteins into the acrosomal granule (Moreno et al. 2000). Throughout acrosome biogenesis, the acrosomal granule is tethered at the center of the acrosomal vesicle. However, very little is known about the possible mechanisms that anchor and maintain the central position of this granule. Interestingly, our recent results have shown that in MYO6-deficient spermatids, the central localization of acrosomal granules is lost in almost a quarter of Snell’s waltzer spermatids (Zakrzewski et al. 2020a). In some of the cells, the granule is completely detached from the inner acrosomal membrane and appears to “float” freely inside the acrosomal vesicle or is even absent. Similar anomalies have been observed in other mouse mutants that lack for example the expression of ZPBP1 (Zona pellucida binding protein 1); (Lin et al. 2007). More severe deformities, such as acrosomal granules, attached ectopically to the outer acrosomal membrane have been described in *Dpy19l2*^*−/−*^ (probable C-mannosyltransferase-null) male mice (Pierre et al. 2012). Uneven distribution of acrosomal material has also been noted in sperm populations lacking the expression of proprotein convertase subtilisin/kexin type 4 (PCSK4); (Tardif et al. 2012). Finally, acrosome malformations have also been reported in *Acrbp*^−/−^ (acrosin-binding protein-deficient) male mice completely lacking an acrosomal granule (Kanemori et al. 2016). The acrosomal defects reported for these mouse mutants may be caused by the destabilization of the multi-layered structure of the acrosome, the improper compaction of the acrosome or the altered processing of the acrosomal proteins (Lin et al. 2007; Pierre et al. 2012; Tardif et al. 2012; Kanemori et al. 2016).

At present, we can only speculate what causes the docking defect of the acrosomal granule in Snell’s waltzer mice as acrosome asymmetry is the only significant change linked to acrosome development in MYO6-deficient spermatids (Zakrzewski et al. 2020a). MYO6 together with its binding partner TOM1/L2 localizes to the acroplaxome right below the acrosomal granule (Zakrzewski et al. 2020a). In MYO6-deficient spermatids, TOM1/L2 is still present at the acroplaxome, which may suggest that MYO6 is not required for the localization of TOM1/L2, but is the essential factor that determines the correct localization of the acrosomal granule within the acrosome. Although the molecular details of how the MYO6-TOM1/L2 complex maintains the symmetrical localization of the acrosomal granule during acrosome biogenesis are unknown, MYO6 may bind via its motor domain to actin filaments in the acroplaxome and through the tail domain to TOM1/L2 (Zakrzewski et al. 2020a). At the same time, TOM1/L2 may interact with a transmembrane protein of the inner acrosome membrane, which in turn may bind with its luminal domain to proteins of the acrosomal granule. This would provide a mechanism for docking of the acrosomal vesicles across the inner acrosomal membrane and a TOM1/L2-MYO6 complex to the underlying actin filaments of the acroplaxome. While the TOM1/L2 interactome has not been established in mouse testes, the three helices in the core of the GAT domain of TOM1/L2 bind to ubiquitin and thus could interact with ubiquitinated transmembrane receptors in the inner acrosome membrane (Wang et al. 2010). Indeed, ubiquitinated proteins have been detected at the acrosomal granule, the inner acrosomal membrane and the acroplaxome, which would support our proposed mechanism of granule docking via the GAT domain of TOM1/L2 and MYO6 binding to actin filaments within the acroplaxome (Haraguchi et al. 2004; Rivkin et al. 2009; Nakamura et al. 2013; Zakrzewski et al. 2020a).

#### Absence of MYO6 affects machinery involved in sperm release in mouse

##### Defects in the spatial organization of endocytic compartment

During spermiogenesis, the maturing spermatids adhere to Sertoli cells and move across the seminiferous epithelium with the help of the apical ES, a unique actin-based and ER-associated anchoring junction (Figs. 1 and 6a). To allow the release of mature spermatozoa into the seminiferous tubules, the apical ES is disassembled and internalized (Adams et al. 2018). A second actin-rich structure is the TBC, which projects from the developing spermatids into the adjacent Sertoli cell cytoplasm and is believed to facilitate the internalization of the apical ES (Figs. 1 and 6e). The structure and molecular composition of TBCs suggests that they are evolutionarily related to endocytic uptake pathways involving clathrin. Indeed, clathrin-coated pits initiate the formation of TBCs and remain at the tip of the extending complexes (Russell and Clermont 1976). Furthermore, the long tubular extensions of TBCs are associated with membrane curvature sensing proteins, such as amphiphysin and dynamin, which facilitates vesicle scission (Kusumi et al. 2007; Vaid et al. 2007). The overall structure of the TBC and the narrow diameter of the tubular extensions is supported by a cuff of dense actin meshwork and several ABPs (Sriram et al. 2016). The bulbar regions of the TBCs and endocytic vesicles internalized from this region are associated with the early endosome marker RAB5 (Young et al. 2012; Adams and Vogl 2017). These endosomes also known as sorting endosomes become positive for EEA1 (early endosome antigen 1) and later recruit LAMP1 (lysosome-associated membrane glycoprotein 1); (Guttman et al. 2004b; Du et al. 2013; Adams and Vogl 2017). Interestingly, our recent results highlight that also MYO6 is localized at the bulbar regions and to vesicles in close proximity to the TBCs (Fig. 6g, h); (Zakrzewski et al. 2020b). Furthermore, we identified two MYO6 adaptor proteins, TOM1/L2 and GIPC1, in a vesicular compartment at the TBCs. These vesicles are positive for APPL1 (adapter protein containing PH domain, PTB domain and leucine zipper motif 1), an adaptor protein present on a subset of RAB5-positive endosomes that are negative for EEA1. It is well established in different cell types and tissues that MYO6 localizes to and tethers RAB5- and APPL1-positive early endosomes to the actin cortex underneath the plasma membrane (Tumbarello et al. 2013; Masters et al. 2017; O’Loughlin et al. 2018). MYO6 maintains the localization of these APPL1-endosomes in the cell periphery, which facilitates maturation and downstream signaling events that precedes cargo processing in EEA1-positive early/sorting endosomes (Tumbarello et al. 2013; Masters et al. 2017). In testes, the lack of MYO6 expression causes the disorganization of the TBCs and loss of spatial integrity of the APPL1-positive early endosomal compartment (Zakrzewski et al. 2020b). These results suggest that MYO6 may stabilize the functional structure of the TBCs by linking TOM1/L2-positive membranes to surrounding actin filaments. After endocytosis from the bulbular region of the TBC, MYO6 tethers and maintains the spatial position of this TOM1/L2-GIPC1 and APPL1-positive endocytic cluster, thus potentially enabling the maturation of this subset of APPL1-containing early endosomes into EEA1-positive endosomes. However, it is still not known whether the spatial integrity of the TBCs is crucial for their function and whether loss of the spatial arrangement of this compartment is directly linked to the slightly reduced number of epididymal sperm and reduced fertility in Snell’s waltzer males (Zakrzewski et al. 2020b). The endocytosis of the junctional protein nectin-3, for example, which forms heterotypic intracellular adhesion junctions at the spermatid/Sertoli cell interface (Rikitake et al. 2012; Adams and Vogl 2017) is not completely blocked in *sv/sv* spermatids. Nectin-3 is still endocytosed and present in vesicular structures in the cytoplasm of *sv/sv* Sertoli cells; however, these vesicles are dispersed and no longer concentrated where the TBCs are clustering (Zakrzewski et al. 2020b). Thus, although the endocytosis of junctional complexes is not entirely inhibited, the dispersion of the TBC endocytic compartment may affect the efficiency of forward trafficking and recycling of nectin-3 to newly formed intercellular attachments in other parts of the Sertoli cell.

Finally, MYO6 is also present together with cortactin and ARP3 at the actin cuffs surrounding the long, extended neck of the TBCs (Zakrzewski et al. 2020b). Interestingly, in Snell’s waltzer mice, these two ABPs are no longer associated with this region of the TBCs, which may suggest a role for MYO6 in the dynamic organization of the actin meshwork surrounding the long proximal tubules of the TBCs. In *Drosophila*, the MYO6 ortholog, jaguar is required for organization of actin cones during spermatid individualization and both ABP, cortactin and ARP3 are displaced from the front of the cones in *jar*^*1*^*/jar*^*1*^ flies (Rogat and Miller 2002; Noguchi et al. 2006). Although the exact role of jaguar in *Drosophila* spermatid individualization is still not known, it seems to facilitate recruitment of different actin regulators to the front of the actin cones, thereby, regulating actin branching through recruitment of the ARP2/3 complex. In mammals, MYO6 has been shown to form a complex with LARG, a RhoGEF that modulates actin organization around endosomes (O’Loughlin et al. 2018) and with DOCK7, also a RhoGEF, which not only regulates actin dynamics but also the assembly of septins structures along actin filaments (Majewski et al. 2012; Sobczak et al. 2016; O’Loughlin et al. 2018). Thus, MYO6 may regulate actin polymerization and organization around the TBCs by interacting with different RhoGEFs.

##### Defects in the structure of the apical ES

The apical ES not only maintains the close contact between maturing spermatids and Sertoli cells but also enables the migration of spermatids across seminiferous epithelium during spermiogenesis and contributes to the positioning of spermatids with their flagella pointed towards the lumen of seminiferous tubules (Dunleavy et al. 2019). The apical ES is a unique anchoring junction consisting of adherens junctions (cadherins/catenins and nectin/afadin complexes), tight junctions (JAM-C and CAR molecules) and focal contacts (α6β1-integrin/laminin α3β3γ3 complex); (Wong et al. 2007; Yan et al. 2008; Kopera et al. 2010). As spermiogenesis progresses and the maturing spermatids move towards the apical compartment of the epithelium, the apical ES undergoes rapid cycles of assembly and disassembly facilitated by the tightly regulated reorganization of actin filaments switching between a bundled and branched configuration. Actin dynamics in the apical ES is regulated by a number of ABPs including for example actin-bundling proteins, such as EPS8, paladin and α-actinin, and proteins regulating branched actin filament assembly, such as the ARP2/3 complex, N-WASP, drebin E and filamin A (Young et al. 2009; Li et al. 2011; Su et al. 2012; Qian et al. 2014a,b; Xiao et al. 2014). Interestingly, the same actin nucleating proteins that control effective actin reorganization in *Drosophila* spermatid individualization—the ARP2/3 complex and cortactin—are also involved in apical ES remodeling (Chapin et al. 2001; Anahara et al. 2006; Lie et al. 2010a). Our analysis using electron microscopy has shown that MYO6 is also present at apical ES during sperm development in mice (Zakrzewski et al. 2017). The signal for MYO6 was predominantly detected in the F-actin bundles of the apical ES that surround the apical pole of the elongating nuclei (Fig. 7a–d). Here, MYO6 may maintain the structural integrity of the apical ES by controlling actin filament dynamics, similar to its role during *Drosophila* spermatid maturation and in other mammalian cell types and tissues (O’Loughlin et al. 2018). Indeed, our preliminary results highlight structural defects, such as swollen ER cisternae and local detachment of the actin bundles from the spermatid head, within the apical ES in MYO6-deficient males (Fig. 8a, b). Interestingly, we did not observe any significant morphological defects in *sv/sv* sperm (except for their slightly reduced number), which would suggest the premature release of round/elongated spermatids to the lumen of seminiferous tubules (Wen et al. 2019; Zakrzewski et al. 2020a). In addition to MYO6, also MYO7a and its binding partner KEAP1 (Kelch-like ECH-associated protein 1), both localize to actin filament bundles within the ES compartment in Sertoli cells. Although MYO7a has been suggested to play a role in spermatid and organelle transport and adhesion during spermatogenesis, the MYO7a KO male mice show no obvious structural disruptions of the apical ES (Hasson et al. 1997; Velichkova et al. 2002; Wen et al. 2019). The knockdown of MYO7a in rat Sertoli cells or in rat testes, however, induced severe disorganization of the actin cytoskeleton across the seminiferous epithelium and abnormal expression of selected ABPs (Wen et al. 2019). Sertoli cell–spermatid adhesion and transport of organelles, such as residual bodies and phagosomes, across the epithelium were grossly disrupted in MYO7a-deficient rat testes, supporting a function for MYO7a in intracellular transport and adhesion during spermiogenesis.

## Perspectives and concluding remarks

The unique reverse directionality of MYO6 may explain why the activity of this motor is necessary at different stages of spermiogenesis and its absence causes a set of specific phenotypes in mice. During the Golgi phase, MYO6 appears to maintain the spatial organization of the Golgi complex and to facilitate a short-distance transport and fusion of proacrosomal vesicles. During the following cap and acrosome phases, depletion of MYO6 impacts on the correct localization of the acrosomal granule and symmetry of developing acrosome. Finally, during the maturation phase, loss of MYO6 disturbs the spatial integrity of the endocytic compartment at the TBC. In addition, our preliminary observations suggest that MYO6 may regulate actin dynamics linked to the apical ES organization. Overall, our observations suggest that in mouse testes, MYO6 plays a structural role during spermatid development by regulating actin dynamics and anchoring different membranous organelles to the surrounding actin cytoskeleton. Although there are certain parallels in the function of MYO6 and its orthologs in regulating actin filament dynamics during *Drosophila* spermatid individualization, *C. elegans* spermatid differentiation, and mouse spermiogenesis, MYO6-deficiency in mouse spermatids is less pronounced and the Snell’s waltzer male mice are only sub-fertile, while MYO6-deficient flies and worms are sterile (Kelleher et al. 2000; Noguchi et al. 2006; Zakrzewski et al. 2020b). Although MYO6 is the only myosin known so far that moves to the minus end of actin filaments, in the opposite direction to all other myosins, it seems surprising that the many different phenotypes uncovered at different stages of spermiogenesis only cause a minor reduction in male mouse fertility of the Snell’s waltzer mice. MYO6, similar to MYO5a and MYO7a, appears to play highly specialized roles at distinct steps of murine spermatid development; however, the lack of single parts of this complex machinery does not completely disrupt the process of mouse spermiogenesis.

While the role of the actin cytoskeleton in mammalian spermiogenesis is a rapidly developing field, our current understanding and recognition of the involvement of different myosins in this process is still less well documented. As highlighted throughout this review, several questions remain, especially in relation to the actin-based origins of acrosome symmetry, regulation of TBC organization and endocytosis, and function of the apical ES in the adhesion of spermatids to Sertoli cells and spermiation. At present, the suggested functions of MYO6 during mouse spermiogenesis are predominantly based on morphological studies of fixed cells and tissues. Although the functional analysis of mammalian spermiogenesis is very complex, future studies may focus on identifying the wider MYO6 interactome in mammalian testis. This would allow us to understand which binding partners and cargo adaptor proteins regulate its function and recruitment to different testicular compartments during sperm development. Moving forward, the use of in vitro Sertoli cell cultures and a combination of CRISPR/Cas9 KO cells with super-resolution confocal microscopy may provide further data to verify the proposed mechanisms of MYO6 function at different phases of spermiogenesis in mammals.
